# Viral loads in throat and anal swabs in children infected with SARS-CoV-2

**DOI:** 10.1080/22221751.2020.1771219

**Published:** 2020-06-09

**Authors:** Chunhui Yuan, Hongmin Zhu, Yuan Yang, Xiaonan Cai, Feiyan Xiang, Huan Wu, Cong Yao, Yun Xiang, Han Xiao

**Affiliations:** a Department of Laboratory Medicine, Wuhan Children’s Hospital, Tongji Medical College, Huazhong University of Science and Technology, Wuhan, People’s Republic of China; b Department of Neurology, Wuhan Children’s Hospital, Tongji Medical College, Huazhong University of Science and Technology, Wuhan, People’s Republic of China; c Institute of Maternal and Child Health, Wuhan Children’s Hospital, Tongji Medical College, Huazhong University of Science and Technology, Wuhan, People’s Republic of China; d Health Care Department, Wuhan Children’s Hospital, Tongji Medical College, Huazhong University of Science and Technology, Wuhan, People’s Republic of China

**Keywords:** SARS-CoV-2, paediatric patients, RT-PCR, diagnostic potential, viral load

## Abstract

Real-time reverse transcriptase-polymerase chain reaction (RT-PCR) assay on anal swabs was recently reported to be persistently positive even after throat testing was negative during severe acute respiratory syndrome coronavirus 2 (SARS-CoV-2) infection. However, data about the consistent performance of RT-PCR assay on throat and anal swabs remain limited in paediatric patients. Here, we retrospectively reviewed RT-PCR-testing results of 212 paediatric patients with suspected SARS-CoV-2 infection at Wuhan Children’s Hospital. The diagnostic potential of these two types of specimens showed significant difference (positive rate: 78.2% on throat swabs vs. 52.6% on anal swabs, McNemar Test *P* = 0.0091) and exhibited a weak positive consistency (Kappa value was 0.311, *P *< 0.0001) in paediatric patients. Furthermore, viral loads detected on both throat and anal swabs also showed no significant difference (*P* = 0.9511) and correlation (Pearson *r* = 0.0434, *P* = 0.8406), and exhibited an inconsistent kinetic change through the course of SARS-CoV-2 infection. Besides, viral loads in the throat and anal swabs were correlated with different types of immune states, immune-reactive phase, and the resolution phase/immunologic tolerance, respectively. These findings revealed that RT-PCR-testing on throat and anal swabs showed significant difference for monitoring SARS-CoV-2 infection and correlated with different immune state in paediatric patients.

## Introduction

There had been 414,179 confirmed cases and 18,440 deaths caused by the ongoing global outbreak of severe acute respiratory syndrome coronavirus 2 (SARS-CoV-2) infection as of March 26, 2020 [1]. Real-time reverse transcriptase-polymerase chain reaction (RT-PCR) assay has been widely used for clinical diagnosis and SARS-CoV-2 has been detected in specimens from multiple sites, including bronchoalveolar lavage fluid, sputum, nasal, anal, and throat swabs of patients with COVID-19 [2]. Although lower respiratory tract samples most often testing positive (93%) for the virus, testing on nasopharyngeal or throat swabs (63–72%) were typically used to confirm the diagnosis.

According to the data collected by Chinese Center for Disease Control and Prevention, 1–2% of Chinese confirmed cases were paediatric patients [3], and more than 90% of paediatric patients were mild or moderate cases [4,5]. The epidemiological and clinical features of children infected with SARS-CoV-2 infection has been reported in our previous study, and about 15.8% laboratory throat swab-testing confirmed cases did not have any symptoms of infection or radiologic features of pneumonia and which were admitted to hospital due to infected family members [6]. Furthermore, several studies have recently reported that anal swabs-testing was persistently positive even after nasopharyngeal testing was negative and maybe more useful in judging the effectiveness of treatment and determining the timing of termination of quarantine [7,8]. Thus, we evaluated the consistency of RT-PCR assay on different types of swabs in paediatric patients through the course of SARS-CoV-2 infection.

## Methods

### Study design and participants

We did a retrospective review of RT-PCR-testing results of 2138 paediatric patients with suspected SARS-CoV-2 infection in Wuhan Children’s Hospital, Huazhong University of Science and Technology (Wuhan, China) from Jan. 1 to Mar. 18, 2020. Wuhan Children’s Hospital is responsible for the treatments of paediatric SARS-CoV-2 infection assigned by the government. Diagnosis of COVID-19 was based on the New Coronavirus Pneumonia Prevention and Control Program (5th edition) published by the National Health Commission of China [9]. All cases were performed RT-PCR assay on throat swabs, and 212 cases were simultaneously tested on anal swabs. 217 cases were tested positive for SARS-CoV-2 either on throat or anal swabs (Fig. S1). Kinetic changes of viral load in both throat and anal swabs were monitored in 13 patients.

This study was reviewed and approved by the Medical Ethical Committee of Wuhan Children’s Hospital, Huazhong University of Science and Technology (approval number IEC-2020R003-E01), and written informed consent was waived because of the retrospective nature of the study. Anonymous clinical data were used for analysis.

### RT-PCR assay

Throat/anal swab samples were collected and tested for SARS-CoV-2 with the Chinese Center for Disease Control and Prevention (CDC) recommended Kit. All samples were processed at the Department of Laboratory Medicine of Wuhan Children’s Hospital. Total RNA was extracted within 2 h using the Nucleic Acid Isolation kit (DAAN Gene, Wuhan, China). The real-time RT-PCR assay was performed using a SARS-CoV-2 nucleic acid detection kit according to the manufacturer’s protocol (BGI Biotechnology, Wuhan, China). Target gene open reading frame 1ab (ORF1ab) was amplified and tested during the real-time RT-PCR assay. A cycle threshold value (Ct-value) in FAM channel ≤38 was defined as a positive test result and a Ct-value of >40 or no amplification curve was defined as a negative test.

### Statistical analysis

We presented continuous variables as median (IQR) or median SD, and categorical variables as number (%). Means for continuous variables were compared using unpaired or paired *t-*tests when the data were normally distributed; otherwise, the Mann–Whitney U test was used. Proportions for categorical variables were compared using the *χ*^2^ test. All statistical analyses were performed using SPSS (Statistical Package for the Social Sciences) version 22.0 software (SPSS Inc). A 2-sided α of less than 0.05 was considered statistically significant.

## Results

### Diagnostic difference of RT-PCR assay on throat and anal swabs

RT-PCR assay on throat and anal swabs were simultaneously tested in 212 of 2138 paediatric patients with suspected SARS-CoV-2 infection in Wuhan Children’s Hospital. 78 of 212 patients were confirmed with SARS-CoV-2 infection according to the positive results obtained from either throat or anal swabs (Table 1). Of the 78 patients, 17 were positive on anal swabs and 37 were positive on throat swabs, as well as 24 were double positive. The diagnostic potential of these two types of specimens showed significant difference (positive rate: 78.2% on throat swabs vs. 52.6% on anal swabs, McNemar Test *P *= 0.0091) and exhibited a weak positive consistency with Kappa value was 0.311 (*P *< 0.0001).

**Table 1.** Diagnostic difference of RT-PCR assay on throat and anal swabs.

		Throat swabs	
		+	−
Anal swabs	+	24	17
	−	37	134

**Figure 1.**
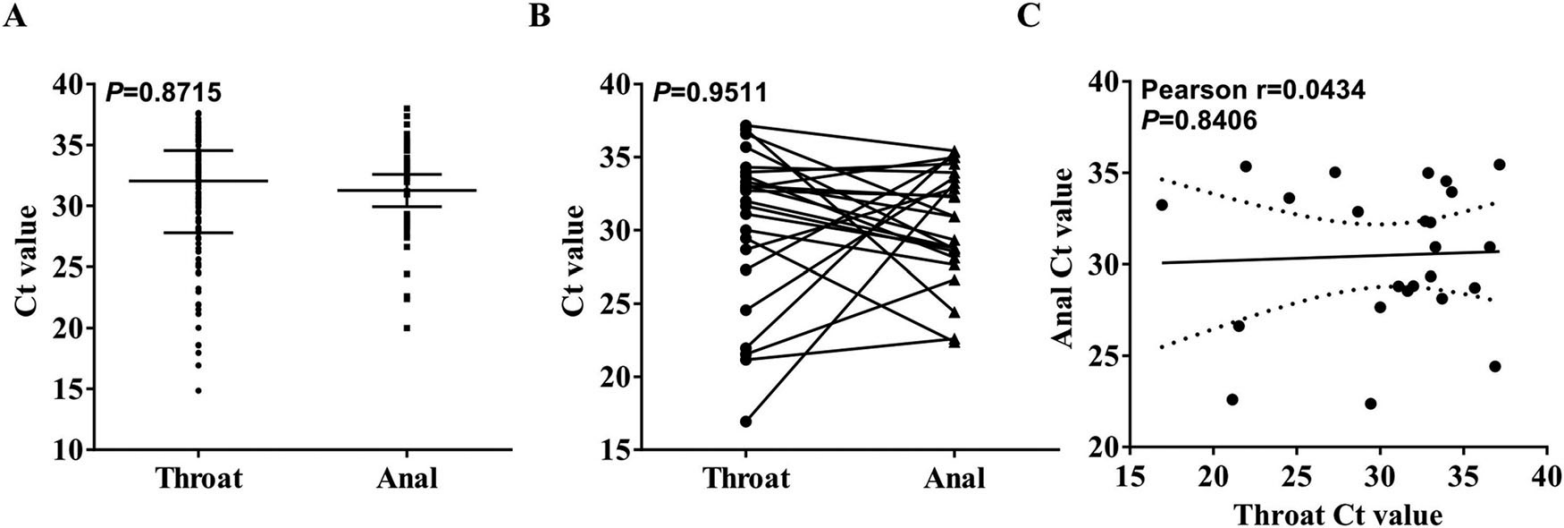
The difference and correlation of Ct value between throat and anal swabs-testing. (A) The difference between Ct value obtained by RT-PCR-testing on throat swabs (200 cases) and anal swabs (41 cases). (B) The difference between Ct value obtained by RT-PCR-testing on paired throat swabs and anal swabs in 24 cases. The data were normally distributed and a paired *t-*test was used to compare statistical differences. (C) The pearson correlation between Ct value obtained by RT-PCR-testing on paired throat swabs and anal swabs in 24 cases.

One thousand nine hundreds twenty-six or 1926 of 2138 cases were only performed RT-PCR assay on throat swabs, and finally 217 cases were tested positive for SARS-CoV-2 either on throat or anal swabs. The viral loads (inversely related to Ct value) between throat swabs (32.05, IQR: 27.80–34.53) and anal swabs (32.28, IQR: 28.63–34.54) showed no difference (Figure 1(A), *P *= 0.8715). In addition, viral loads detected on both throat and anal swabs of 24 patients also showed no significant difference (Figure 1(B), *P *= 0.9511) and correlation (Figure 1(C), Pearson *r* = 0.0434, *P *= 0.8406).

### Correlation of viral loads to indexes of SARS-CoV-2 infection

**Figure 2.**
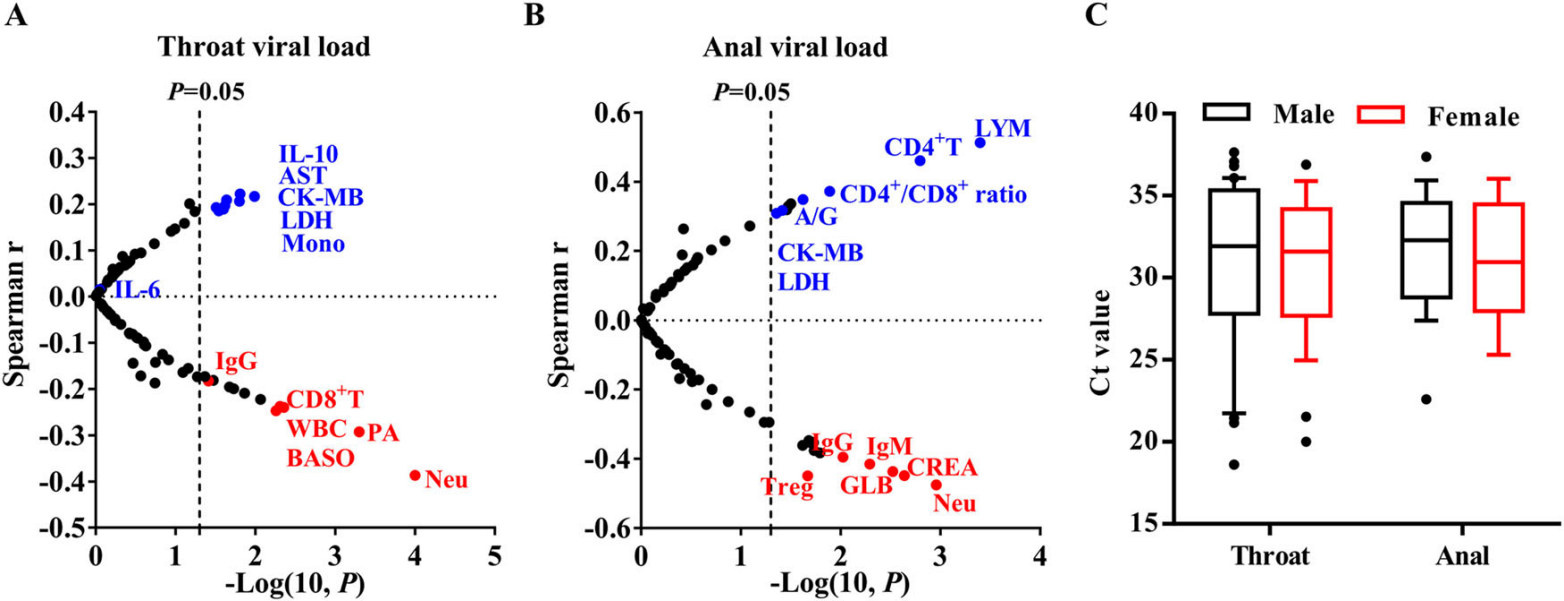
Correlation of viral loads to indexes of SARS-CoV-2 infection. (A) The correlation of viral loads to indexes of SARS-CoV-2 infection. Ct values were detected in throat swabs obtained from 200 cases and viral loads were inversely correlated to Ct value. (B) The correlation of viral loads in anal swabs of 41 cases to indexes of SARS-CoV-2 infection. (C) The difference of Ct value detected in throat and anal swabs between males and females.

The correlations between viral loads with indexes of SARS-CoV-2 infection, including sex, age, blood routine, blood chemistry, infection-related biomarkers (CRP, PCT, and FERR), immunoglobulins, complements, B cells, NK cells, and subsets of T cells were further evaluated. Viral loads in both throat and anal swabs were positively correlated with myocardial zymogram, CK-MB and LDH in paediatric patients, while negatively correlated with neutrophils (Neu) (Figure 2 (A,B)). IL-10 was positively correlated with throat viral loads, whereas CD8^+^ T cells and white blood cells (WBC) showed a negative correlation (Figure 2(A)). Counts of CD4^+^ T cells were positively correlated with anal loads, whereas Tregs, IgG, and IgM were negatively correlated with anal loads (Figure 2(B)). Inflammatory IL-6 levels showed no significant correlation with both throat and anal viral loads in paediatric patients. Also, viral loads in both throat and anal swabs showed no difference between males and females (Figure 2(C)).

### Kinetic changes of viral loads through the course of SARS-CoV-2 infection

**Figure 3.**
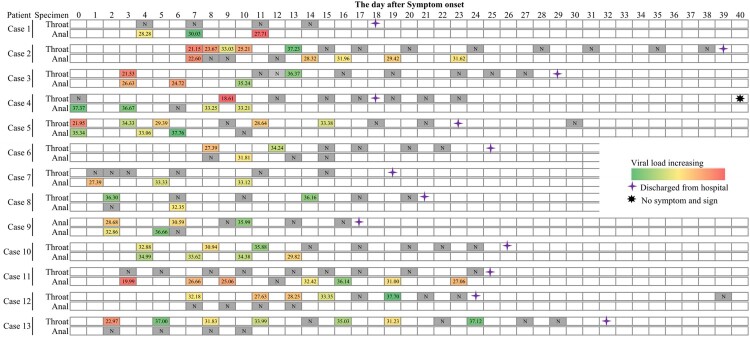
Timeline of RT-PCR-testing results from throat and anal swabs through the course of SARS-CoV-2 infection for 13 cases, January to March 2020.

RT-PCR-testing on specimens obtained for respiratory tract was recommended for judging the time of hospital discharge or discontinuation of quarantine. Thus, dynamic changes of viral load were only detected in both throat and anal swabs of 13 patients concerning the day of confirmation (Figure 3). In our results, we noticed that throat swab-testing was persistently negative in Case 1, Case 7, and Case 11, and anal swab-testing remain relatively higher levels 1–4 days before discharging from hospital. RT-PCR-testing results of Case 12 and Case 13 were persistently negative on anal swabs. The difference of clinical features between with anal single positive result and throat single positive were further investigated, and the results revealed that clinical features, including signs and symptoms, CT scan, underlying diseases, comorbid conditions, hospital stay, and time from positive to negative for PCR assay showed no significant difference (Table 2).

**Table 2.** Difference of clinical features between patients with anal single positive result and throat single positive.

	Anal single positive (*n* = 17)	Throat single positive (*n* = 37)	*χ*^2^	*P*-value
**Sex (n. %)**			0.17	0.4089
Male	11 (64.7)	26 (70.3)		
Female	6 (35.3)	11 (29.7)		
**Age (m, IQR)**	74 (32–100)	90 (39–140)		0.4055
**Onset of symptom to Hospital admission (d, IQR)**	10 (7–12)	5 (3–10)		0.0184
**Signs and symptoms (n. %)**			3.36	0.0668
None	10 (58.8)	12 (32.4)		
Yes	7 (41.2)	25 (67.6)		
**Gastrointestinal symptoms (n. %)**				0.3035
None	11 (64.7)	30 (81.1)		
Yes	6 (35.3)	7 (18.9)		
**Cough (n. %)**			1.19	0.1635
None	13 (76.5)	21 (56.8)		
Yes	4 (23.5)	16 (43.2)		
**Fever (n. %)**			2.85	0.0917
None	12 (70.6)	17 (45.9)		
Yes	5 (29.4)	20 (54.1)		
**CT evidence of pneumonia (n. %)**			1.19	0.5507
None	7 (41.2)	12 (32.4)		
Unilateral	8 (47.1)	16 (43.3)		
Bilateral	2 (11.7)	9 (24.3)		
**Underlying diseases (n. %)**				0.7027
None	15 (88.2)	30 (81.1)		
Yes	2 (11.8)	7 (18.9)		
**Comorbid conditions (n. %)**				0.3653
None	14 (82.4)	34 (91.9)		
Yes	3 (17.6)	3 (8.1)		
**Hospital stay (d, IQR)**	13 (11–15)	16 (10–20)		0.4573
**Time from positive to negative for PCR assay (d, IQR)**	6 (4–10)	7 (5–14)		0.2354

## Discussion

As the only centre assigned by the central government for treating infected children under 16 years of age in Wuhan, we presented the inconsistent viral presentation of RT-PCR assay on throat and anal swabs for monitoring paediatric SARS-CoV-2 infection in this study. This report, to our knowledge, is the largest case series to date for evaluating the monitoring performance of paediatric patients with SARS-CoV-2 infection.

According to the study conducted by Gong et al in 10 paediatric patients, they reported that testing on anal swabs remained detectable well even after throat testing was negative and suggested the possibility of faecal-oral transmission besides respiratory transmission [8]. Cell entry of SARS-CoV-2 virus depends on the binding of the viral spike (S) proteins to cellular receptor ACE2 and S protein priming by host cell protease TMPRSS2 [10]. Lung type II pneumocytes, nasal goblet secretory cells, and ileal absorptive enterocytes are highly susceptive to SARS-CoV2 infection due to simultaneously expression of ACE2 and TMPRSS2 [11,12]. In contrary to the report that infectious virus could not be isolated from stool samples conducted by Wendter et al. [13], Li et al recently isolated live SARS-CoV2 virus from faecal samples in 3 of 11 adult patients, and the potential reason may be the diverse mutations in S proteins of the virus isolated from an individual patient [14]. In our study, we found that viral loads showed no difference and correlation between throat and anal swabs. Furthermore, the clinical features between anal single positive result and throat single positive showed no significant difference. Thus, similar to SARS-CoV and MERS coronavirus [15], faecal-oral transmission is also one route for SARS-CoV-2 infection.

CD4^+^ T helps cells limit exhaustion of memory CD8^+^ cytotoxic T lymphocyte (CTL) during influenza A virus infection [16], and Tregs were most important for calming inflammation during the resolution phase of viral infection [17]. Huang et al reported that faecal samples from 33 (45%) of 74 patients were negative for SARS CoV-2 RNA whereas their respiratory swabs were positive from first symptom onset, and positive results obtained from faecal samples were delayed from throat swabs through the course of 41 patients with SARS-CoV-2 infection [7]. Although we did not conduct strictly paired time-course analysis, we noticed that some cases were persistently negative in throat swabs and others were persistently negative in anal swabs. Thus, we suspect that increased viral loads in throat swabs may represent an acute infection (immune-overreactive) phase of SARS-CoV-2 infection and predict the exhaustion of memory CTL, which frequently occurred in patients associated with acute respiratory distress syndrome [18]. No matter what the transmission potential of patients with anal or faecal positive results, decreased viral loads in anal swabs may indicate the resolution phase of SARS-CoV-2 infection and has the potential to be used for monitoring the effectiveness of therapeutic responses as which negatively correlated with Tregs, IgM, and IgG.

In conclusion, RT-PCR-testing on throat and anal swabs showed significant difference for monitoring SARS-CoV-2 infection and correlated with different immune states in paediatric patients. Although testing on anal swabs or faecal samples was not included in guidelines for New Coronavirus Pneumonia Prevention and Control Program [19], it has caused attention to the decision on when to discontinue precautions to prevent transmission [20]. Transmission potential of anal swabs-testing positive patients, especially children, which are more like to be asymptomatic carriers, need be urgently evaluated for the control of outbreak SARS-CoV-2 infection.
